# Does enrollment status in community-based insurance lead to poorer quality of care? Evidence from Burkina Faso

**DOI:** 10.1186/1475-9276-12-31

**Published:** 2013-05-16

**Authors:** Paul Jacob Robyn, Till Bärnighausen, Aurélia Souares, Germain Savadogo, Brice Bicaba, Ali Sié, Rainer Sauerborn

**Affiliations:** 1 Institute of Public Health, University of Heidelberg, Heidelberg, Germany; 2 The World Bank, 1818 H Street NW, Washington, DC, USA; 3 Department of Global Health and Population, Harvard School of Public Health, Boston, USA; 4 Africa Centre for Health and Population Studies, University of KwaZulu-Natal, Mtubatuba, South Africa; 5 Nouna Health Research Centre, Ministry of Health, Nouna, Burkina Faso; 6 Nouna Health District, Ministry of Health, Ouagadougou, Burkina Faso

**Keywords:** Community-based health insurance, Health insurance, Health care providers, Quality of health care, Health care utilization, Burkina Faso

## Abstract

**Introduction:**

In 2004, a community-based health insurance (CBI) scheme was introduced in Nouna health district, Burkina Faso, with the objective of improving financial access to high quality health services. We investigate the role of CBI enrollment in the quality of care provided at primary-care facilities in Nouna district, and measure differences in objective and perceived quality of care and patient satisfaction between enrolled and non-enrolled populations who visit the facilities.

**Methods:**

We interviewed a systematic random sample of 398 patients after their visit to one of the thirteen primary-care facilities contracted with the scheme; 34% (n = 135) of the patients were currently enrolled in the CBI scheme. We assessed objective quality of care as consultation, diagnostic and counselling tasks performed by providers during outpatient visits, perceived quality of care as patient evaluations of the structures and processes of service delivery, and overall patient satisfaction. Two-sample t-tests were performed for group comparison and ordinal logistic regression (OLR) analysis was used to estimate the association between CBI enrollment and overall patient satisfaction.

**Results:**

Objective quality of care evaluations show that CBI enrollees received substantially less comprehensive care for outpatient services than non-enrollees. In contrast, CBI enrollment was positively associated with overall patient satisfaction (aOR = 1.51, p = 0.014), controlling for potential confounders such as patient socio-economic status, illness symptoms, history of illness and characteristics of care received.

**Conclusions:**

CBI patients perceived better quality of care, while objectively receiving worse quality of care, compared to patients who were not enrolled in CBI. Systematic differences in quality of care expectations between CBI enrollees and non-enrollees may explain this finding. One factor influencing quality of care may be the type of provider payment used by the CBI scheme, which has been identified as a leading factor in reducing provider motivation to deliver high quality care to CBI enrollees in previous studies. Based on this study, it is unlikely that perceived quality of care and patient satisfaction explain the low CBI enrollment rates in this community.

## Introduction

Good quality of care is an important objective for health sectors in developing countries for a variety of reasons. Quality of care lies on the pathway from health systems activities to health outcomes and overall patient satisfaction. From the perspective of patient rights, patients from all socio-economic levels who seek healthcare deserve correct and courteous treatment, safe medical conditions, and sufficient information on their health status and treatment options [1-3]. It has also been argued that providing high quality services can lead to increased health service utilization and, in turn, reduce unsupervised and often risky self-treatment [4-6].

Research on quality of care in developing countries has continued to increase over the past two decades [7]. Formal sector services that are often evaluated include family planning [1], primary care [7,8], and reproductive health [9]. Primary-care quality assessments commonly include a variety of tools, such as patient-provider direct observation surveys (assessing the clinical or technical quality of care), facility assessment surveys (measuring the structural quality of care), provider interview surveys (measuring provider competency, clinical knowledge and professional background), and patient-perspective surveys (assessing perceived quality of care and overall patient satisfaction) [10-12]. Studies that investigate quality of care from the patient perspective have collected information through exit interviews [8], mystery clients [13], household surveys [14,15], and focus groups [16,17]. Studies on perceived quality of care predominantly measure perceptions among people who actually visit health facilities, often using the resulting information as a basis for designing interventions to improve patient satisfaction [8,18-20].

Community-based health insurance (CBI), one form of community financing, has been seen as an attractive solution to the challenge of generating financial resources for the formal health sector in developing countries [21-24]. In particular, it is a potential instrument to improve access to health care by reducing financial barriers to health services, empowering enrollees through fostering dialogue between communities and health care providers, and improving quality of care by introducing contractual arrangements contingent on quality standards [25-32]. In recent years, the development of CBI programs in sub-Saharan Africa has garnered substantial interest by both researchers and policymakers alike. Currently, data on quality of care for patients who enroll in community-based health insurance (CBI) schemes is very limited [21,24]. Evidence on the relationship between health insurance and quality of care in sub-Saharan Africa is scarce, although a recent systematic review concluded that there was a weakly positive effect of both social health insurance and CBI on quality of care [33].

In early 2004, a community-based health insurance (CBI) scheme was introduced in Nouna district, Burkina Faso. The details of the implementation of the CBI scheme and the benefit package have been described elsewhere [34-36]. Primary- and secondary-care facilities that operate within the CBI implementation zone signed two-year contracts with the insurance scheme, in which the method and schedule for provider payments for coverage of enrollees’ expenses were defined. In May 2010, when the study was conducted, all thirteen primary-care facilities and the one secondary-care facility (the district hospital) within the zone in which the CBI has been implemented had contracted with the scheme. These facilities were paid by the CBI on an annual capitation basis, i.e., the facilities received a flat payment per individual enrolled in the CBI. Payments were only intended to cover the cost of drugs prescribed to enrollees by health care providers.

Enrollment rates in the Nouna CBI scheme have remained low compared to anticipated rates of close to 50%, despite an upward trend over time [37,38]. During the first year of operation (2004) the patient enrollment rate was 5%, but it increased to 9% by 2010. The enrollee drop-out rate has substantially declined over time but remains considerable; it was 32% in 2004 and 16% in 2010. In 2006, the most common reasons for dropping out, after affordability of the insurance premium (28%), involved patient dissatisfaction with the quality of care provided to CBI enrollees. Patients judged the quality of care to be poor regarding both the health services they received, such as drugs, as well as medical staff behavior [39].

A recent mixed methods study on the relationship between CBI provider payment and health worker satisfaction in Nouna found that insufficient level of capitation payments, infrequent schedule of capitation payment, and lack of a payment mechanism for reimbursing service fees were the payment attributes that most strongly affected provider satisfaction. It is plausible that poor health worker satisfaction with CBI provider payment has translated into a quality of care differential between CBI enrollees and non-enrollees [40].

Assessments of quality of diagnostic consultations are often limited to indicators such as consultation time or patient evaluations of particular attributes of the structures and processes of care delivery, while professionally defined quality indicators linked to clinical actions remain rare. The quality of diagnostic care in rural Burkina Faso has previously been identified as low, even potentially dangerous to patients [41]. This article uses exit interview data to assess potential differences between CBI enrollees and the general population not enrolled in the scheme in the objective and subjective quality of care provided by primary-care facilities contracted with the Nouna CBI scheme. Specifically, we investigated differences between enrolled and non-enrolled patients in (i) the clinical comprehensiveness of diagnostic care provided during outpatient consultations, (ii) perceptions on structures and processes of service delivery, and (iii) overall patient satisfaction with health services received. Understanding patient perspectives on quality of care can assist policymakers in improving patient satisfaction and health care utilization [18]. Differences in quality of care between CBI enrollees and other patients may inform CBI reform, with the aim of ensuring appropriate health care utilization among enrollees and expanding CBI coverage to people who are currently not enrolled.

## Methods

### Study setting

The study took place in the Nouna health district in northwest Burkina Faso, a predominantly rural area where the majority of the population depends on subsistence agriculture as their primary livelihood [42,43]. The city of Nouna, approximately 300 km from Ouagadougou (the capital of Burkina Faso) and approximately 100 km from the border with Mali, is both the headquarters of Nouna health district and the administrative center of the province of Kossi. At the time of the study, a total of thirty-four primary-care facilities in Nouna district, staffed by certified nurses and trained midwives, were providing basic outpatient and maternity services. Thirteen of these facilities are located within the intervention zone of the Nouna CBI scheme.

### Study design and data collection

We conducted a cross-sectional survey of 398 patients seeking outpatient consultations at the district’s thirteen primary-care facilities that contracted with the CBI scheme. CBI enrollees are entitled to comprehensive outpatients services. Sample size calculations indicated that a minimum of 25 patients would need to be interviewed per facility to ensure sufficient statistical power. We employed systematic random sampling to select patients for exit interviews among all the patients seeking general outpatient services over the study period. On a randomly selected facility day, we started with a randomly chosen first patient (e.g., the fifth patient presenting to the facility). We then sampled every third patient following the initially selected patient. On average, approximately 3–5 patients were interviewed per day. Patients visiting the facilities for other types of care, such as pre-natal consultations or institutional deliveries, were not included in the sample. Because CBI enrollees must be referred by a contracted primary-care facility in order to be covered for care at the district hospital, patients visiting outpatient services at the district hospital were also not included in the study. From April 25th to May 20th, 2010, we collected data by conducting exit interviews with patients after they had completed their visit and departed from the facility grounds. We interviewed the patients after they had given informed, written consent. If a patient was less than 15 years of age, the adult accompanying them participated in the interview. Interviews were conducted by field workers recruited and trained by Nouna Health Research Center.

### Questionnaire

The questionnaire included five sections: (1) patient identity, (2) socio-economic characteristics, (3) illness symptoms and care prior to seeking care at a primary-care facility, (4) outpatient consultation and actual diagnostic services received during the visit, (5) patient perceptions on the quality of structures and processes of service delivery, and (6) overall patient satisfaction with treatment received (Table 1 and 2). After translating and back-translating the questionnaire in the four primary local languages (Dioula, Bwamou, Mooré, Fulfuldé), the questionnaire was pre-tested on 30 patients.

**Table 1:** Objective quality of care indicators

**Objective quality of care: consultation and diagnostic care**	
Provider weighed the patient	Provider asked the patient aboutthe history of the illness
Provider took the temperature of the patient	Provider asked about the patient’s symptoms
Provider used a stethoscope	Provider asked if treatment was taken before arrival at the facility
Provider examined the patient (touch stomach, ears, throat, etc.)	Provider explained to the patient the diagnosis
Provider asked to see the patient’s health card	Provider informed the patient about CBI enrollment/renewal

**Table 2:** Indicators of perceived quality of care by domain

**Indicators of perceived quality of care**	
**Domain 1: Perceived availability of health care providers, supplies, and physical resources**	**Domain 4: Perceived financial and physical accessibility to care**
Medical supplies and equipment are sufficient	Alternative payment options are available
Rooms are sufficient	The cost of services is manageable
Adequate/appropriate health care providers for women	The cost of prescribed drugs is manageable
There is sufficient high quality health care providers	Distance to the facility is accessible
Medicine for all illnesses is always available	Health care providers give sufficient time to their patients
**Domain 2: Perceived quality of health care delivery**	**Domain 5: Perceived quality of physical structure of facility**
Health care providers conduct quality diagnostic exams	Health facility is clean and orderly
Health care providers make appropriate drug prescriptions	Easy to identify location of specific services at facility
The quality of drugs prescribed is good	Patients feel comfortable and safe while waiting
Treatment provided is efficient and effective	
**Domain 3: Perceived quality of health care provider conduct**	
Health care providers show compassion and support for patients	
Health care providers are respectful to patients	
Health care providers provide quality follow-up care	
Health care providers are welcoming during consultations	
Health care providers respect patient confidentiality	
Facility assistants are friendly and helpful to patients	
Facility assistants respond to patients questions	

#### Checklist of diagnostic services and care patient received

Diagnostic quality of care was assessed by asking patients about the activities the health care providers performed during consultation services for outpatient visits. Patient exit interviews were chosen as a method as opposed to direct observations of patient-provider interactions to minimize the influence of the observers on the health workers’ activities (i.e. Hawthorne effects) [44]. Patients were asked if the health worker who provided the consultation performed the following actions: weighing the patient, taking the patient’s temperature, using a stethoscope, physical examination (touching stomach, ears, throat, etc.), reviewing the patient’s personal health card, asking about the history of the illness, asking about the patient’s symptoms, asking if the patient sought or received treatment prior to visiting the facility, and explaining to the patient the diagnosis. Patients were also asked if the health worker mentioned enrollment in the CBI scheme, including questions about re-enrollment for current enrollees or new enrollment for non-enrolled patients.

#### Perceived quality of structures and processes of service delivery

This section on perceived quality of care was based on a measurement scale developed and validated in previous studies in Guinea [45] and in Nouna district, Burkina Faso [7,8]. Prior to the use in Nouna district, the instrument was adapted by an exploratory qualitative study in the same community [46]. For our study, the final instrument included 24 items for quality assessment. These items can be grouped into five domains of perceived quality of care: (1) perceived availability of health care providers, supplies, and physical resources, (2) perceived quality of health care delivery, (3) perceived quality of health care provider conduct, (4) perceived financial and physical accessibility of care, and (5) perceived quality of physical structure of facility. Respondents could express their perceived quality of care on a six-level Likert scale: very poor (1), poor (2), somewhat poor (3), somewhat good (4), good (5), and very good (6). The respondents were asked their opinion about the services they received the day they were interviewed. For each respondent, summary scores, or “overall patient satisfaction” scores were calculated by summing individual quality scores for each item. Once the aggregate or “overall patient satisfaction” score was calculated, the distribution of scores were fit into six quantiles to create a six-level ordinal scale for “overall patient satisfaction” with levels ranging from very poor (1) to very good (6).

### Ordinal logistic regression model

Given the ordinal quality of the six-level outcome variable “overall patient satisfaction”, ordinal logistic regression was used to assess the relationship between this outcome and key respondent characteristics. The proportional odds model (also known as cumulative logit model) is an appropriate method of analysis when one is presented with a grouped continuous response variable, because it provides a single estimate of the log odds ratio over the cut-off points, allowing for ease of interpretation of the data and in terms of model parsimony [47].

Within the context of the study’s “overall patient satisfaction” scale (*y*), let *Y* denote the response and *y*_*1*_, *y*_*2*_, *y*_*3*_, *y*_*4*_, *y*_*5*_, and *y*_*6*_ the categories of the q of care score: “very poor” (1); “poor” (2); “somewhat poor” (3); “somewhat good” (4); “good” (5); and “very good” (6), respectively. In this case, five “cut-points” (*T*_*1*_*, T*_*2*_, *T*_*3*_, *T*_*4*_, *T*_*5*_) separate the six levels of *y*. Thus Pr (*Y* = *y*_*ij*_) is the probability that a randomly selected individual *i* is in category *j*. The ordinal response categories are monotonically related to an underlying continuous latent variable y*. For one independent variable (χ) the structural model is *y* = *α* + *βχ* + ϵ . The standard formula for the predicted probability in the ordinal regression model is denoted as:

$$\mathrm{Pr}\left(y=\left(m\right|\chi \right)=F\left(\tau_{m}-\chi \beta \right)-F\left(\tau_{m-1}-\chi \beta \right)$$

where *F* is the cumulative distribution function (cdf) for *ϵ*[48].

There is general consensus that the assumptions underlying the proportional odds approach are quite stringent, in particular when one considers more than one covariate [47,49]. Thus, we applied a Wald test of the proportional-odds assumption to check the assumption of proportionality for the final model.

The explanatory variable of primary interest was CBI coverage status; i.e., an indicator variable capturing CB enrollment at the time of the visit. Other socio-demographic and treatment characteristics were included in the model as explanatory variables. Socio-demographic variables included age (continuous), sex (male = 1, female = 0), whether the respondent had ever been to school or not (yes = 1, no = 0), religion (Muslim = 1, other = 0), and residential zone (urban = 1, rural = 0). Illness and treatment history variables included whether symptoms at onset of illness included febrile symptoms or not (fever = 1, other = 0), whether the illness aggravated prior to seeking facility-based care (yes = 1, no = 0), and whether the patient sought other types of care prior to seeking facility-based care (yes = 1, no = 0). Characteristics for the primary-care facility treatment they received on the day of the interview included perceived waiting time (classified into five categories, from very short = 1 to very long = 5), total cost of care (for service fees and drugs), whether the provider who treated them was the facility head nurse or not (yes = 1, no = 0), and whether the provider had informed the patient about the diagnosis of illness (yes = 1, no = 0).

### Ethics

The University of Heidelberg received approval for the research from their human subjects committee in Germany (130/2002) which was extended in 2005 and 2008, as well as the Nouna Health Research Center ethical committee (2005-005/CLE/CRSN). All respondents were informed of the research objectives and were asked to take part in the study. Those who agreed were asked to sign a consent form.

## Results

Interviews were conducted with 398 patients visiting primary-care facilities, with 99% consent rate to be interviewed (9 patients refused to be interviewed). As shown in Table 3, 135 patients interviewed (34%) were enrolled in CBI at the time of the interview. Fifty-nine percent of patients were male, and the median age was 19 years of age (SD = 19). Seventy-five percent of people accompanying child patients were the patient’s mothers, and 78% of patients over 14 years of age were married. Fifty-six percent of respondents were Muslim, and 63% of respondents had never been to school. Only one respondent had completed secondary education. The majority of respondents were farmers; a large proportion of respondents were involved in small trade throughout the year, often in addition to farm work (Table 3).

**Table 3:** Socio-demographic characteristics of participants

**Socio-economic characteristics**	**Value**	
	**No.**	**%**
Respondents	398	100
**Sex**		
Male	176	58.5
Female	222	41.5
**Age**		
< 1	53	13.3
1-4	86	21.6
5-14	70	17.6
15-24	52	13.1
25-34	59	14.8
35-44	23	5.8
45-54	35	8.8
55-64	10	2.5
65+	10	2.5
**CBI**^**1 **^**enrollment status of patient (n = 398)**		
Currently enrolled at time of consultation (2010)	135	33.9
**Person who accompanied children under 15 (n = 209)**		
Mother	157	75.1
Father	34	16.3
Grandmother	2	1.0
None	4	1.9
Other	12	5.7
**Marital situation of patients aged 15+ (n = 189)**		
Single	34	18.0
Married	148	78.3
Separated/divorced/widowed	7	3.7
**Religion of respondent (n = 398)**		
Muslim	221	55.5
Catholic	126	31.7
Protestant	38	9.5
Animist	11	2.8
No religion	2	0.5
**Education level reached of respondent (n = 398)**		
None	249	62.6
Primary incomplete	59	14.8
Primary complete	41	10.3
Secondary incomplete	48	12.1
Secondary complete or higher	1	0.3
**Dry season economic activity of respondent (n = 398)**		
None	114	28.6
Small commerce	235	59.0
Migration to city	4	1.0
Animal husbandry	7	1.8
Artisanal work	5	1.3
Other	33	8.3
**Rainy season source of revenue of respondent (n = 398)**		
None	153	38.4
Small commerce	189	47.5
Remittance	21	5.3
Business enterprise	7	1.8
Salary	2	0.5
Retirement pension	1	0.3
Other	25	6.3
**Illness and treatment-seeking history (n = 398)**		
First illness symptom: fever	152	38.2
First illness symptom: headache	42	10.6
First illness symptom: stomach ache	21	5.3
First illness symptom: cough	18	4.5
Illness aggravated before seeking facility care	298	74.9
Received household treatment before seeking assistance	138	34.7

^1^ CBI, Community-based insurance. No.: Number.

### Objective quality of care

Table 4 summarizes information related to the characteristics and the objective quality of care received on the day of the interview. While there was no significant difference in the number of days between onset of illness symptoms and seeking facility care (p = 0.277), the reason for visiting the primary-care facility differed significantly between the two groups (p < 0.001). The primary reason for the uninsured was “the nature of the illness” (31%); the most common reason among the insured was “enrolled in CBI” (38%). There were substantial differences in the cost of care between the two groups, particularly for service fee payments (p < 0.001), the cost of drugs (p < 0.001) and the total cost of care (p < 0.001).

**Table 4:** Characteristics and objective quality of care by insurance status

**Reason for visit, cost of care, diagnostic indicators**	**Uninsured**		**Insured**		**Test statistic**	**P-value**
**Reason for visit**	**Number**	**%**	**Number**	**%**	**Pearson X**^**2**^	
Nature of illness	81	31.2	19	14.1	107.037	<0.001
Enrolled in CBI^a^	4	1.5	52	38.5		
Appreciation for health care provider’s quality of care	61	23.5	29	21.5		
Advice from friend/relative	42	16.2	9	6.7		
Close proximity	27	10.4	12	8.9		
Confidence in modern medicine	39	15.0	14	10.4		
Other	6	2.3	0	0.0		
**Days between onset of illness symptoms and seeking facility care**	**Number**	**%**	**Number**	**%**	**Pearson X**^**2**^	
0	19	0.1	3	0.0	21.056	0.277
1-2	109	0.4	59	0.4		
3-5	87	0.3	48	0.4		
5+	45	0.2	25	0.2		
**Health care provider who consulted the patient**	**Number**	**%**	**Number**	**%**	**Pearson X**^**2**^	
Head nurse	40	15.4	14	10.4	2.4544	0.293
Other provider (auxiliary nurse, auxiliary midwife, etc.)	219	84.2	121	89.6		
Don’t know	1	0.4	0	0.0		
**Cost of care (FCFA**^**b**^**)**	**Mean**	**SD**	**Mean**	**SD**	**T-test**	
Cost of service fee	119.4	102.6	0.0	0.0	13.464	<0.001
Cost of medication/drugs	1029.4	1065.7	18.1	148.8	10.964	<0.001
Other (parking, etc.)	6.5	13.9	3.7	10.8	2.065	0.0395
Total cost	1149.4	1089.2	9.6	111.9	12.118	<0.001
**Consultation and diagnostic care (yes = 1, no = 0)**	**Number**	**%**	**Number**	**%**	**Z-test**	
Did the provider weigh the patient	41	15.8%	34	25.0%	2.596	0.009
Did the provider take the temperature of the patient	205	78.1%	65	48.3%	3.087	0.002
Did the provider use a stethoscope	104	39.6%	59	44.0%	2.725	0.007
Did the provider examine the patient (touch stomach, ears, throat, etc.)	125	47.7%	65	48.5%	2.029	0.043
Did the provider ask to see the patient’s health card	210	80.0%	30	22.3%	−3.986	<0.001
Did the provider ask the patient about the history of the illness	249	94.6%	26	19.0%	−0.739	0.460
Did the provider ask about the patient’s symptoms	249	94.6%	20	14.8%	−1.469	0.143
Did the provider ask if treatment was taken before arrival at the facility	167	63.5%	62	46.2%	−1.223	0.222
Did the provider explain to the patient their illness	57	21.5%	44	32.4%	2.376	0.018
Did the provider inform the patient about CBI enrollment/renewal	21	8.1%	23	17.0%	1.985	0.048

^a^ CBI: Community-based health insurance.

^b^ FCFA: Franc Communauté Financière Africaine, the local currency used in Burkina Faso. 500 FCFA = $1 USD.

SD: Standard deviation. For each indicator, a two-group variance test for equal variance was conducted.

There were also significant differences in objective quality of care. Overall, the diagnostic care provided to CBI enrollees was significantly less comprehensive than care provided to non-enrollees. Health care providers were less likely to perform the following actions on insured patients than on uninsured patients: measure weight (p = 0.009), take temperature (p = 0.002), use a stethoscope (p = 0.007), physically examine the patient (p = 0.043), and inform the patient diagnostic results (p = 0.018).

### Perceived quality of care

Table 5 presents how enrolled and non-enrolled patients perceived the quality of different structures and processes of service delivery at the health facility. On average, both insured and uninsured patients were most satisfied with health care workers’ respect for patient confidentiality, with no significant difference between the two groups. Both groups of patients were least satisfied with the availability of alternative options for payments.

**Table 5:** Perceived quality of care by insurance status

**Indicators of perceived quality of care**	**Non-insured**		**Insured**		**t-test**	**p-value**
	**Mean**	**SD**	**Mean**	**SD**		
**Perceived availability of health care providers, supplies, and physical resources**						
Medical supplies and equipment are sufficient	5.3	0.8	5.2	0.7	−0.671	0.503
Rooms are sufficient	4.8	0.7	4.9	0.7	−0.522	0.602
Health care providers are appropriate for women	4.5	0.9	4.8	0.9	−2.375	0.018
There are sufficient high-quality health care providers	5.0	0.7	5.1	0.7	−1.706	0.089
Medicine for all illnesses is always available	4.3	1.3	4.2	1.1	0.195	0.838
**Perceived quality of health care delivery**						
Health care providers conduct quality diagnostic exams	5.3	0.8	5.2	0.7	2.123	0.034
Health care providers make appropriate drug prescriptions	5.0	0.5	4.9	0.6	0.767	0.444
The quality of drugs prescribed is good	4.9	0.6	4.9	0.7	1.152	0.250
Treatment provided is efficient and effective	4.6	0.7	4.7	0.8	−0.172	0.863
**Perceived quality of health care provider conduct**						
Health care providers show compassion and support for patients	4.8	1.0	4.7	0.8	0.214	0.831
Health care providers are respectful to patients	5.2	0.9	5.3	0.7	−1.490	0.137
Health care providers provide quality follow-up care	4.7	0.7	4.6	0.8	1.391	0.165
Health care providers are welcoming during consultations	5.4	0.7	5.3	0.7	1.740	0.083
Health care providers respect patient confidentiality	5.6	0.6	5.5	0.7	1.922	0.055
Facility assistants are friendly and helpful to patients	5.0	0.7	5.2	0.7	−2.556	0.011
Facility assistants respond to patients questions	5.4	0.7	5.6	0.5	−3.069	0.002
**Perceived financial and physical accessibility to care**						
Alternative payment options are available	2.9	1.6	2.9	1.7	−0.086	0.931
The cost of services is manageable	4.4	0.7	4.6	0.7	−3.629	<0.001
The cost of prescribed drugs is manageable	4.2	0.8	4.6	0.7	−4.094	<0.001
Distance to the facility is accessible	3.2	3.0	2.9	1.7	1.283	0.200
Health care providers give sufficient time to their patients	5.4	0.7	5.2	0.8	2.300	0.022
**Perceived quality of physical structure of facility**						
Health facility is clean and orderly	5.5	0.7	5.4	0.7	1.673	0.095
Easy to identify location of specific services at facility	4.8	0.6	4.9	0.6	−0.581	0.562
Patients feel comfortable and safe while waiting	5.2	0.8	5.1	0.7	0.191	0.849
**Overall patient satisfaction**^1^	115.2	9.2	115.5	7.2	−0.427	0.670

^1^ Overall patient satisfaction: Aggregate sum of individual scores for indicators of perceived quality of care. Each indicator was scored on a scale of 1 (very poor) to 6 (very good).

SD: Standard deviation. For each indicator, a two-group variance test for equal variance was conducted.

There were significant differences in perceived quality of care between the two groups for several indicators. The insured group had higher mean scores for the following indicators: health care providers are appropriate for women (p = 0.018), facility assistants are friendly (p = 0.011), facility assistants respond to patient questions (p = 0.002), the cost of services is manageable (p < 0.001), and the cost of prescribed drugs is manageable (p < 0.001). The non-insured group had higher mean scores for two indicators: health care providers conduct quality diagnostic exams (p = 0.034) and health care providers provide sufficient time to patients (p = 0.022).

#### Determinants of overall patient satisfaction

To assess determinants of overall patient satisfaction with quality of care, ordinal logistic regression analysis was performed. As the final model passed the Wald test of the proportional odds assumption (p < 0.245) the proportional odds model was used. The results are shown in Table 6. As indicated in the table, CBI enrollment had a significant and positive impact on the overall patient satisfaction with quality of care (aOR = 1.51, p = 0.014). Illness aggravation prior to facility care was significant and had a negative impact on overall patient satisfaction (aOR = 0.45, p = 0.018). Shorter perceived waiting times had a significant positive impact (aOR = 1.63, p = 0.002), while residing in an urban zone (Nouna town) had a significant negative impact overall patient satisfaction with quality of care (aOR = 0.27, p = 0.024). Education level, febrile symptoms at onset of illness, sex, age, religion, total cost of care, the provider being the facility head nurse, and the provider informing the patient of the illness diagnosis had no significant impact on overall patient satisfaction.

**Table 6:** Ordinal logistic regression results on the determinants of overall patient satisfaction

**Variable**	**aOR**	**s.e.**	**P-value**	**95% CI**	
Age (continuous)	1.003	0.006	0.617	0.992	1.014
**Enrolled in CBI**^**a **^**(yes/no)**	**1.513**	**0.254**	**0.014**	**1.088**	**2.102**
Ever been to school (yes/no)	1.560	0.556	0.212	0.776	3.136
First illness symptoms (fever = 1, other = 0)	1.558	0.585	0.242	0.747	3.250
Illness aggravated before deciding to seek facility care (yes/no)	0.452	0.151	0.018	0.235	0.871
Delay > 2 days occurred before seeking facility care (yes/no)	0.755	0.106	0.045	0.574	0.994
Total cost of care (consultation, drugs, other) (FCFA^b^)	0.999	0.000	0.543	1.000	1.000
Perceived waiting time (1 = very long, 5 = very short)	1.625	0.252	0.002	1.199	2.203
Provider was the head nurse of the facility (yes/no)	1.359	0.342	0.223	0.830	2.226
Provider informed the patient of the diagnosis (yes/no)	3.410	2.560	0.102	0.783	14.850
Male (yes/no)	0.865	0.147	0.395	0.619	1.208
Urban (yes/no)	0.267	0.182	0.024	0.085	0.842
/cut1	−1.027	0.344		−1.701	−0.352
/cut2	0.297	0.120		0.061	0.533
/cut3	1.056	0.158		0.746	1.366
/cut4	1.802	0.465		0.891	2.712
/cut5	3.133	0.476		2.200	4.066
**No. of respondents**	**398**				
**Pseudo R-squared**	**10.930**				

^a^ CBI: Community-based health insurance, aOR = adjusted odds ratio, s.e. = standard error, CI = confidence interval.

^b^ FCFA: Franc Communauté Financière Africaine, the local currency used in Burkina Faso. 500 FCFA = $1 USD.

## Discussion

We find that within the context of the Nouna CBI scheme in Burkina Faso, there were significant differences between insured and uninsured patients in several indicators of perceived quality of care. In particular, CBI enrollees perceived quality to be higher regarding the appropriateness of health care for women, friendliness and availability of facility assistants, and financial accessibility to services and drugs. Only with regards to one indicator of perceived quality of care – the perceived quality of diagnostic exams – did the uninsured patients perceive quality to be higher. In regression analyses, CBI enrollment had a significant positive impact on overall patient satisfaction.

### Objective quality of care

In contrast to these findings on quality perceptions, CBI enrollees scored lower on objective quality of care indicators. They received substantially less comprehensive care for consultation and diagnostic services: providers were less likely to weigh, take the temperature, perform a physical examination, use a stethoscope, and inform patients about the diagnosis of their illness, when the patients were enrolled in the CBI. As one of the primary objectives of the CBI scheme is to improve access to quality health services among the enrolled population, these findings suggest an unintended consequence of the CBI insurance reform. Our findings corroborate and quantify previous qualitative data on comprehensiveness of care that patients received before and after enrolling in CBI in this community [16]. We explore potential factors that may be driving these differences below.

### Perceived adequacy of resources and services

In Nouna district, patients visiting the various health centers tended to positively evaluate the availability of providers and physical health care resources, particularly with regards to medical supplies and equipment. CBI enrollees were more likely to perceive that the available providers were appropriate for women (for example, female health workers providing maternal health services to women), but there was no difference in the quality perceptions about the availability of medicines. This result is in conflict with earlier publications that highlighted CBI enrollees dissatisfaction with availability of drugs at primary-care facilities, as well as providers’ claims of drug stock ruptures due to elevated demand for prescribed drugs by CBI enrollees [16,17,39]. This divergence from earlier findings may be due to several factors. First, in recent years the Ministry of Health and Nouna district medical team has introduced policies to reduce the frequency and duration of drug stock-outs, improving the general availability of drugs in primary care health facilities. Second, over time providers may have improved their specific prescription patterns for CBI enrollees, leading to more comprehensive provision of drugs for patients enrolled in the scheme. Such potential behavior changes over time after CBI introduction require further research, but it seems plausible that providers change their behaviors as they experience the consequences of CBI on their clinical practice and learn to improve their interactions with the CBI administration.

### Perceived quality of health care delivery

Both groups of patients gave high ratings on the quality of health care delivery. While this finding is similar to a previous study on health care provision in Nouna [8], it is contrary to results from other studies in this community, in which physical examination, diagnosis and prescription were seen to be inadequate by respondents [7,41,50]. In our study, only one indicator related to diagnostic quality was significantly different between the two groups. CBI enrollees were found to view the quality of diagnostic exams lower than non-enrollees. This difference is in line with our results that show lower quality of care for CBI enrollees, where we found that CBI enrollees were less likely to have received the basic services that all patients should receive during diagnostic consultations. While our study found no significant difference between the two groups on other indicators for health care delivery, previous studies had found that CBI enrollees often feel that health care providers do not provide appropriate or sufficient drug prescriptions to enrollees, leading to lower levels of efficacy in treatment for patients enrolled in CBI [15,17,39]. One plausible explanation for this result is that CBI enrollees may have lowered their expectations regarding the quality of care they will receive over time. During the first few years of CBI operations health workers were providing poorer care to CBI enrollees to protest the new provider payment methods introduced by CBI [16,40]. As a result, CBI enrollees’ quality of care expectations might have changed, leading to the current subjective assessments of quality of care.

### Perceived quality of health care provider conduct

Both the insured and the uninsured patients perceived the conduct of their health care providers to be very good, a finding which is similar to results from one previous quality of care study in Nouna district [7], but contrary to another one [8], in which indicators related to provider conduct were rated very poorly. Past studies suggested that CBI enrollees’ poor perception of their health care providers was an important reason for dropping out of the Nouna CBI scheme [16,17,39], yet in this study no significant differences on perceptions of health care providers’ conduct were found between the two groups, with the exception of two indicators related to facility assistants. These findings may be due to changes in the relationship between the CBI scheme and health care providers since the abovementioned studies were conducted, leading to potential improvements in the relationship between health care providers and the CBI management structure or increased health worker support for the insurance scheme. Alternatively, it is possible that over time those CBI enrollees who were dissatisfied with the conduct of their health care providers selectively left the CBI, so that those people who are still enrolled are on average more satisfied with their providers.

### Perceived financial and physical accessibility to care

Study participants generally gave low ratings to the affordability and accessibility of care. These results confirm findings from earlier publications on quality of care in Nouna district [7,8]. Surprisingly there was no significant difference between the CBI enrollment groups for these two indicators, which is contrary to results from previous qualitative studies on the Nouna scheme [16,17]. Our study found that lack of access to credit remained a problem for both groups. Given that the CBI benefit package does not cover certain services such as those related to maternity care or treatment of chronic diseases [35,51], CBI enrollees may still face financial barriers for certain types of care. They may also not be well informed on the types of care covered through enrollment. Lack of understanding of the CBI benefit package in Nouna district has been identified as a residual barrier to appropriate care for CBI enrollees [16]. Enrollees have been found to seek care at contracted facilities, only to find that the appropriate treatment for the specific illness is not covered by the CBI benefit package. If short-term credit is not available and enrollees arrive at facilities without cash on hand to pay for services or drugs, this may lead to poor perceptions of the facility visited.

### Perceived quality of physical structure of facility

Indicators for physical structure of the facility received relatively high remarks, which is similar to results identified by quality of care studies in Latin America [1] and Nepal [9]. Structural elements of health service delivery, such as the cleanliness and physical appearance of health facilities, have been identified as driving factors in patients’ perceptions on service quality and overall satisfaction [52]. No significant differences were found between CBI enrollees and non-enrollees, suggesting that differences in perceived quality of care and overall patient satisfaction among the two groups is primarily driven by the process of service delivery and less by physical characteristics of where the services are delivered.

### Possible explanations for differences in quality of care between CBI enrollees and non-enrollees

Prior research on the Nouna CBI scheme using mixed methods data [40], qualitative data [16], and data from discrete choice experiments [53] found wide-spread health-worker dissatisfaction with the CBI payment methods. The sources of dissatisfaction included (i) the low overall level of capitation; (ii) the payment schedule (once per year in July); (iii) the removal of patient user fees , and (iv) the fact that capitation was the only payment mechanism used by the CBI (when additional payment mechanisms could possibly improve health-worker motivation and the financial situation of facilities). The differences in quality of care between enrolled and non-enrolled patients may have been caused by the unintended consequences of the changes in provider payment methods. Capitation payment has the advantage that it incentivizes health care providers to improve efficiency of health care delivery and minimize treatment costs; a well-known disadvantage is that it may lead to lower quality of care [54]. Since health care providers received capitation payment for the care of CBI enrollees, but service fees for the care of non-enrollees, it is possible that the lower quality of the care delivered to CBI enrollees is a direct effect of the payment method [53].

However, there are alternative or additional explanations for the differential outcomes in quality of care. There may be selection effects influencing the observed outcomes, as enrolled patients may be systematically different to non-enrolled patients and these underlying differences might explain the observed differences in quality of care. For example, socio-demographic differences between CBI enrollees and non-enrollees, such as age and socio-economic status, might lead to differential treatment by health workers. Another explanation may be that the provision of care to enrolled patients involves additional administrative work, which may affect providers’ attitudes towards CBI patients and time available for patient care.

There may also be systematic differences in expected quality of care between the enrolled and non-enrolled populations, with different anchoring points influencing perceptions on quality of care. It is plausible that certain aspects of patients’ experiences with receiving health services may be driving the observed differences in perceived quality of care more so than others. In particular financial access to health services and drugs, which was rated substantially higher among enrolled patients than non-enrolled patients, may outweigh the influence of other aspects of care received, such as time spent with providers or comprehensiveness of diagnostic exams. When financial access to health services improves, other components of health service delivery related to the diagnostic quality of care may become relatively less important in influencing patients’ overall satisfaction with the care they received.

One of our primary results – that CBI enrollment is associated with higher perceived quality of care – differs significantly from prior studies of the Nouna CBI scheme. As several years have passed since these earlier studies were conducted and published, the relationship between health care providers and CBI enrollees may have improved. One significant change since 2008 is the introduction of a formal contract signed between the CBI scheme and the district health office, with the intention to both strengthen the relationship between the two parties as well as clarify best practices for providing care to CBI enrollees.

While little is known about the relationship between quality of care and insurance status in developing countries [33], in other contexts lack of insurance has been associated with poorer quality of care [55,56], but also with better quality of care [57]. Our findings in Burkina Faso suggest that insurance enrollment can in fact lead to poorer objective quality of care. A principal factor for this outcome may be cost-cutting incentives for health workers generated by the type and level of provider payment method used by the scheme [40,53].

### Study limitations

Our study has several potential limitations. As noted in previous studies, patients tend to state that they are overall highly satisfied with the quality of care they have received; however, satisfaction with particular, concrete dimensions of quality of care is commonly low [7,8,45,58,59]. For instance, a recent study in Madagascar found that patient satisfaction was overall high but low regarding facility condition and supplies [58]. While the discrepancy between overall and component satisfaction is not implausible, it could also be the consequence of survey biases, such as social desirability bias or cognitive difficulties in evaluation complex phenomena. We tried to eliminate sources of social desirability bias by explaining clearly to respondents that all of their answers were anonymous and would not be shared with either health care providers or people associated with the CBI scheme, but we cannot rule at that these measures were incompletely effective.

It is also possible that the survey itself biased the study findings because it could have induced behavior changes among the health care providers – awareness of being observed might change the behavior of the observed – but since the providers were not aware of the study objective this type of observation bias may not have been severe. Even if health workers had changed the quality of care in response to being observed, it seems unlikely that that would have changed quality of care differentially by patients’ CBI enrollment.

We have identified a strong relationship between CBI enrollment status and quality of care. The strength of our inferences regarding causality of the observed relationship is increased by the fact that we have qualitative findings that suggest that providers are likely to treat CBI patients differently due to the incentives generated by the CBI payment method [40]. The qualitative findings show that providers not only delivered less comprehensive care to CBI enrollees (because they feared bankruptcy and were generally dissatisfied with the CBI payment method). A quantitative approach to strengthen our inferences regarding causality of the observed relationship between CBI enrollment status and quality of care would be to instrument CBI enrollment using a variable that significantly determines CBI enrollment but does not independently determine quality of care. Unfortunately, we could not identify such an instrumental variable, because at the time of the study, CBI was universally offered to the entire population throughout the intervention zone in Nouna district and the approach through which CBI was offered was highly standardized and did not differ across individuals by any factor we could observe.

## Conclusions

Recognizing quality shortcomings in the provision of health care services in developing countries has motivated efforts to measure and monitor service quality via surveys of health care providers and their clients [59,60]. Within the context of the Nouna CBI scheme, patient satisfaction with the quality of care received under CBI enrollment plays an important role in the decision to re-enroll [16,39]. We find that there were significant differences in perceived quality between enrolled and non-enrolled patients, and that overall patient satisfaction was higher among enrollees. In contrast to this finding, however, CBI enrollees received care that was *objectively* of worse quality. This result may be an effect of the type of provider payment used by the scheme (capitation) which may lead to provider rationing of services and reduced health worker motivation to deliver high quality care. While the primary objective of the CBI scheme was to improve access of the local population to quality health services, the program enrollees have in fact received care of poorer quality than patients who were not enrolled in the scheme. In order to improve the quality of care for CBI enrollees, one possible solution may thus be to revise provider payment methods to align incentives of health workers with one of the key objectives of community-based insurance: to improve access to comprehensive, high quality health services. This objective could potentially be achieved by (i) increasing capitation payment rates to ensure that providers receive sufficient resources to deliver comprehensive services to CBI enrollees, (ii) introducing direct financial incentives to providers based on the quantity or quality of services provided to enrollees, or (iii) introducing bonus payments to health facilities linked to enrollment outcomes in facility catchment areas.

## Competing interests

The authors declare that they have no competing interests.

## Authors’ contributions

PR was the principal investigator, analyzed the data, and wrote the paper. TB made substantial contributions to the study design (methods) interpretation of the results. He also reviewed the first draft. ASO, GS, BB, ASI, and RS contributed significantly to management of the study throughout the data collection process. RS made substantial contributions to the study design and development of the research question. All authors read and approved the final manuscript.
